# A Pragmatic Intervention Using Financial Incentives for Pregnancy Weight Management: Feasibility Randomized Controlled Trial

**DOI:** 10.2196/30578

**Published:** 2021-12-24

**Authors:** Rebecca Krukowski, Brandi Johnson, Hyeonju Kim, Saunak Sen, Riad Homsi

**Affiliations:** 1 Department of Preventive Medicine College of Medicine University of Tennessee Health Science Center Memphis, TN United States; 2 Department of Public Health Science University of Virginia Charlottesville, VA United States; 3 Just For Women Obstetric Clinic Memphis, TN United States

**Keywords:** pregnancy, weight, physical activity, self-weighing

## Abstract

**Background:**

Excessive gestational weight gain (GWG) is common and can result in maternal and child health complications. Pragmatic behavioral interventions that can be incorporated into standard obstetric care are needed, and financial incentives are a promising approach.

**Objective:**

The aim of this study is to evaluate the feasibility of recruitment, randomization, and retention, as well as treatment engagement and intervention satisfaction, in a behavioral program. The program provided small incentives for meeting behavioral goals of self-weighing and physical activity as well as larger outcome incentives for meeting GWG goals.

**Methods:**

We recruited 40 adult women in their first trimester of pregnancy from February 2019 to September 2019 at an obstetric clinic. Participants were randomized to 3 intervention components using a 2×2×2 factorial design: daily incentives for self-weighing (lottery vs certain loss), incentives for adhering to the Institute of Medicine’s GWG guidelines based on BMI category (monthly vs overall), and incentives for reaching physical activity goals (yes vs no). Participants were asked to complete daily weigh-ins using the Withings Body wireless scale provided by the study, as well as wear a physical activity tracker (Fitbit Flex 2). Feasibility outcomes of recruitment, randomization, and retention, as well as treatment engagement and intervention satisfaction, were assessed. Weight assessments were conducted at baseline, 32-week gestation, and 36-week gestation.

**Results:**

Participants were enrolled at, on average, 9.6 (SD 1.8) weeks’ gestation. Of the 39 participants who were oriented to their condition and received the intervention, 24 (62%) were Black or African American, 30 (77%) were not married, and 29 (74%) had an annual household income of less than US $50,000. Of the 39 participants, 35 (90%) completed the follow-up data collection visit. Participants were generally quite positive about the intervention components, with a particular emphasis on the helpfulness of, and the enjoyment of using, the e-scale in both the quantitative and qualitative feedback. Participants who received the loss incentive, on average, had 2.86 times as many days of self-weighing as those who received the lottery incentive. Participants had a relatively low level of activity, with no difference between those who received a physical activity incentive and those who did not.

**Conclusions:**

A financial incentive–based pragmatic intervention was feasible and acceptable for pregnant women for promoting self-weighing, physical activity, and healthy GWG. Participants were successfully recruited early in their first trimester of pregnancy and retained for follow-up data collection in the third trimester. Participants demonstrated promising engagement in self-weighing, particularly with loss-based incentives, and reported finding the self-weighing especially helpful. This study supports further investigation of pragmatic, clinic-based financial incentive–based interventions for healthy GWG behaviors.

**Trial Registration:**

ClinicalTrials.gov NCT03834194; https://clinicaltrials.gov/ct2/show/NCT03834194

## Introduction

### Background

Excessive gestational weight gain (GWG) increases risk for high-cost obstetric conditions such as labor complications and gestational diabetes mellitus for mothers in the short term [1-4]. Excessive GWG also has long-term risks for maternal weight retention [5-7] and childhood obesity [7-9]. For these reasons, GWG is a serious public health concern, particularly because 38% of the women with normal weight, 62% of the women with overweight, and 56% of the women with obesity exceed the GWG guidelines of the Institute of Medicine (IOM) [10]. Pragmatic behavioral interventions that can be incorporated into standard obstetric care are needed.

Financial incentives have been used alone or in combination with other interventions to improve a variety of health outcomes [11-14], including in weight management [15-19]. In addition, incentives are an effective strategy to facilitate smoking cessation among pregnant women [20,21], and incentives may be more attractive for the circumscribed pregnancy period rather than, for example, weight maintenance, which has a much longer time horizon. Surprisingly, however, only 1 study has examined the effect of financial incentives on meeting GWG recommendations; this study found that providing incentives did not increase adherence to GWG outcomes [22]. However, experts have recommended using incentives to encourage health behaviors rather than outcomes [23].

Behaviors that negatively affect health often involve immediate benefits and delayed costs. For example, *eating for two* provides immediate gratification but may lead to excessive GWG [24]. In contrast, behaviors associated with successful weight management (such as daily self-weighing [25-29], weight goals [30], and exercise goals [25,30-33]) often involve immediate time costs with delayed and uncertain health benefits [24]. Thus, incentivizing more immediate GWG-related behaviors may be better than just incentivizing longer-term outcomes.

### Types of Incentives

Incentives, however, can vary widely in their certainty, format, and frequency of distribution [15]. It is generally thought that incentives are more likely to be effective when they are framed as avoiding losses rather than making gains [34] and when rewards are provided immediately [35]. Others have also recommended using small but frequent incentives because these incentives are more visible than large but infrequent payouts [24,36]. A recent study found support for the loss aversion framework in increasing step goal achievement; a greater proportion of participants achieved the daily step goal when it was framed as a loss compared with when the incentive was framed as a gain or a lottery [37]. Another study found that participants who received a lottery-based incentive for reaching a weight loss goal had greater success than the control group [18]. Recent research has also found enhanced engagement and greater weight loss among individuals who received both behavior- and outcome-focused incentives compared with those who did not receive incentives [16,38]. Thus, it will be important to determine what types of incentives are most promising for encouraging GWG-related behaviors and outcomes.

Consistent with previous research and based on self-regulation theory principles [39], this study examines the impact of small incentives for meeting behavioral goals of self-weighing and physical activity as well as larger outcome incentives for meeting GWG goals. The aim of this pilot study is to evaluate the feasibility of recruitment, randomization, and retention, as well as treatment engagement and intervention satisfaction.

## Methods

### Study Design

This pilot study recruited participants from February 2019 to September 2019 from an obstetric clinic with 1 physician in Memphis, Tennessee. Participants were randomized to 3 intervention components using a 2×2×2 factorial design: (1) daily incentives for self-weighing on a wireless scale (lottery vs certain loss), (2) incentives for adhering to the IOM’s GWG guidelines based on BMI category (monthly vs overall), and (3) incentives for reaching physical activity goals (yes vs no). The intervention lasted approximately 6 months, depending on participants’ gestational age at baseline. Assessments were conducted at baseline, 32-week gestation, and 36-week gestation (if the participant had not yet delivered). Participants were asked to complete daily weigh-ins using the Withings Body wireless scale provided by the study, as well as wear a physical activity tracker (Fitbit Flex 2; Google LLC). Incentives (US $30 for each follow-up visit) were used to facilitate high retention at both follow-up data collection visits, which took place at the obstetric clinic. This study was registered with ClinicalTrials.gov (NCT03834194) and approved by the institutional review board of the University of Tennessee Health Science Center.

### Sample

Potential participants were identified by the clinic’s nurses and obstetrician at their pregnancy confirmation visit or self-identified through recruitment materials posted at the obstetric clinic (including in the examination rooms and waiting room; Multimedia Appendix 1). They were encouraged to meet with the study team in the obstetric office if they wished to learn more about the study and potentially enroll in the study. Interested individuals were evaluated for the eligibility criteria, and electronic informed consent was obtained. The obstetric clinic has approximately 16 pregnancy confirmation visits per month.

To be eligible, individuals needed to be aged at least 18 years and no more than 13 weeks’, 0 days’ gestation upon recruitment (based on the date of their last menstrual period and then confirmed by their physician at their first prenatal visit) because GWG-focused interventions that begin in the first trimester are more effective [40]. Individuals also needed to verify that they were having a singleton pregnancy by ultrasonography because of the different GWG guidelines for women with multiple gestation [7], and participants were enrolled only if the physician believed that the intervention would be safe for them. Additional eligibility criteria included (1) having a BMI of ≥18.5 kg/m^2^ (because of the infrequency of women with underweight) and (2) having access to wireless internet or a Bluetooth-connected device to facilitate data collection for participants’ self-weighing behavior and physical activity.

### Enrollment

A total of 40 participants provided informed consent and were enrolled on the web using the Way to Health web platform developed by the University of Pennsylvania, which integrated enrollment, randomization, surveys, automated delivery of study email messages, and transfer of data from the study’s wireless devices [41]. All participants received a Fitbit Flex 2 and a Withings Body scale at enrollment to track their physical activity and weight over time. Before randomization, research staff members oriented the participants to the devices (including providing handouts to review if questions arose later). The staff members then assisted participants with setting up Fitbit and Withings accounts, downloading the apps to their smartphones, pairing the devices with Bluetooth, and authorizing the transfer of data to the Way to Health platform. Participants were instructed to open the apps each day to transmit their data to the Way to Health platform.

### Randomization

After enrollment, participants were randomized into 1 of 8 conditions (Table 1), that represented all combinations of the 3 different components. Participants received an intervention orientation message and handout that detailed the components to which they were randomized, the recommendation to weigh daily, and a GWG recommendation tailored to BMI category (with overall or monthly goals). They also received a handout that provided strategies for achieving healthy GWG (Multimedia Appendix 2).

**Table 1:** Randomized conditions (N=40).

Condition	Self-weighing	Weight goal	Physical activity goal	Participants, n (%)
1	Lottery	Monthly	No	5 (13)
2	Lottery	Overall	Yes	5 (13)
3	Lottery	Overall	No	5 (13)
4	Lottery	Monthly	Yes	5 (13)
5	Loss	Monthly	No	5 (13)
6	Loss	Overall	Yes	5 (13)
7	Loss	Overall	No	5 (13)
8	Loss	Monthly	Yes	5 (13)

### Procedures

#### Self-weighing Lottery-Based Incentive

Participants randomized to a condition with the self-weighing lottery-based incentive (conditions 1, 2, 3, and 4) were asked to pick a *lucky* number from 0 to 99 at randomization. They were informed that there would be a daily lottery for which they would be eligible if they weighed themselves on the previous day on the scale provided by the study team. Then, for each day of their pregnancy, participants were informed of the study’s randomly generated winning lottery number. Participants who weighed themselves on the previous day and who had a 1- or 2-digit match between their *lucky* number and the number that was drawn were notified of their reward, consistent with previous research [42]. A 2-digit match (1 in 100 chance) yielded a US $15 incentive and a single-digit match (1 in 5 chance) yielded a US $2 incentive in the form of an Amazon gift card. Participants who did not weigh themselves the previous day were informed that they could have won incentives, consistent with loss aversion principles [43] (see Multimedia Appendix 3 for sample email messages). Participants also received automated email messages when they did not transmit data, reminding them to self-weigh and sync their devices. The daily winnings were accumulated for the week starting Monday and were disbursed the following Monday. The maximum payout for this component was US $112 in total.

#### Self-weighing Loss-Based Incentive

Participants randomized to a condition with the self-weighing loss-based component (conditions 5, 6, 7, and 8) had a weekly balance of US $3.50 at the beginning of each week in their account. Then, for each day that they did not weigh, US $0.50 was subtracted from this account. Participants who did not weigh themselves the previous day were informed that they had lost US $0.50, consistent with loss aversion principles [43] (see Multimedia Appendix 3 for sample email messages). The daily winnings were accumulated for the week starting Monday and were disbursed the following Monday. The maximum payout for this component was US $112 total.

#### Monthly GWG Goal Incentive

Participants randomized to a condition with the monthly GWG goal component (conditions 1, 4, 5, and 8) received US $14 per month if their monthly GWG was within the recommended monthly range for their BMI category (Table 2), which they had received in a handout at randomization. Participants received a monthly email regarding whether their GWG was within the recommended range or not and, thus, whether they received the monthly GWG incentive (see Multimedia Appendix 3 for sample email messages).

The monthly GWG goals were constructed based on the IOM’s recommended range of total GWG tailored to BMI category, the minimum recommended GWG in the first trimester for all BMI categories (1.1 lb), and the range of recommended weekly GWG in the second and third trimesters by BMI category [7]. Participants were eligible for the incentive starting from the first full month of pregnancy after the start of their participation. To receive the incentive, the participant needed to self-weigh on the study-provided scale on at least 1 day in the last week of the gestational month as well as have at least one weight in the previous month to calculate GWG within the month.

**Table 2:** Monthly weight gain goals by weight status^a^.

			Monthly weight gain goals (lb)				
			Normal weight		Overweight		Obese
**First trimester**							
	Weeks 5-8	<2.2		<2.2		<2.2	
	Weeks 9-12	<2.2		<2.2		<2.2	
**Second trimester**							
	Weeks 13-16	2.8-5		1.4-3.6		0.8-2.8	
	Weeks 17-20	2.8-5		1.4-3.6		0.8-2.8	
	Weeks 21-24	2.8-5		1.4-3.6		0.8-2.8	
**Third trimester**							
	Weeks 25-28	2.8-5		1.4-3.6		0.8-2.8	
	Weeks 29-32	2.8-5		1.4-3.6		0.8-2.8	
	Weeks 33-36	2.8-5		1.4-3.6		0.8-2.8	

^a^There was no lower bound for the first trimester weight goal so as to not penalize pregnant women who have trouble gaining weight because of nausea.

#### Overall GWG Goal Incentive

Participants randomized to a condition with the overall GWG goal component (conditions 2, 3, 6, and 7) were provided the IOM’s overall GWG recommendation based on their BMI at randomization (Table 3). The overall GWG goals were constructed based on the IOM’s recommended range of total GWG and the mean of recommended weekly GWG in the second and third trimesters. These values were adjusted for 8 weeks (for data collection at 32 weeks) and 4 weeks (for data collection at 36 weeks) from a full-term pregnancy at 40 weeks. If the participant met the goal, she received the overall GWG incentive after the 36-week visit, unless she delivered before this visit (in which case she received the incentive based on her 32-week visit weight upon notification that she had given birth).

**Table 3:** Overall weight gain goals by weight status (adjusted goals based on data collection at 32 weeks’ and 36 weeks’ gestation).

Gestational weeks	Overall weight gain goals (lb)		
	Normal weight	Overweight	Obese
32	17-27	10.2-20.2	7-16
36	21-31	12.6-22.6	9-18

#### Weekly Physical Activity Goal Incentive

Participants randomized to an arm with the physical activity incentive component (conditions 2, 4, 6, and 8) were encouraged to achieve a goal of 150 minutes of physical activity per week based on the guideline from the American College of Obstetricians and Gynecologists [44]. Participants received US $3.50 if they met the activity goal each week. *Fairly active minutes* and *very active minutes* based on the Fitbit activity tracker programming counted toward the 150-minute goal [45]. Participants who did not meet the activity goal were notified that they would have received US $3.50 had they met their activity goal (see Multimedia Appendix 3 for sample email messages). The maximum payout for this component was US $105 (US $3.50 per week for a participant who joined the study at 6 weeks’ gestation).

### Measures

#### Sociodemographic Characteristics

At baseline, participants reported their age, race, ethnicity (Hispanic or non-Hispanic), marital status, educational background, employment status, income, and the number of children in their household.

#### Participant Recruitment

Recruitment yields were calculated based on the number of participants who indicated interest in the study and the number of participants who were randomized.

#### Perceptions About the Effectiveness of the Incentive Conditions

The baseline assessments included participants’ perceptions about which of the incentive types would be more effective (daily self-weighing: lottery vs loss-based; weight gain goals: monthly vs overall).

#### Intervention Satisfaction

At the 32-week follow-up assessment, participants were asked about their satisfaction with the intervention. Specifically, they were asked to indicate how satisfied they were with each component of the intervention on a 5-point scale from 1 (not at all) to 5 (extremely), including an option to indicate *not applicable* if they had not received the component. They were asked to separately rate the helpfulness and enjoyment aspect of the emails, electronic scales, and Fitbit activity trackers. They were also asked to separately rate the usefulness and enjoyment aspect of the lottery incentive, loss incentive, the monthly weight gain goal, overall weight gain goal, and the weekly physical activity goal. In addition, they were asked to rate how likely it was that they would recommend using an electronic scale, a fitness tracker, setting a goal for GWG, and setting a goal for physical activity for managing GWG to a friend. Finally, at the end of the structured questionnaire, participants were asked to respond to 3 open-ended questions regarding the recommendations they would make for changes to the program, aspects of the program that were most helpful, and anything else that they wanted to share that they thought might help improve pregnancy weight management programs in the future.

#### Intervention Engagement

Participants’ daily self-weighing behavior (coded each day as present or absent) and weekly physical activity (measured in active minutes) were monitored electronically because they were measures of treatment engagement.

#### Retention

Program retention was observed from the number of participants who consented to participate and then completed the follow-up data collection visits at 32 weeks’ and 36 weeks’ gestation. Participants were alerted that they would be withdrawn from the study if they exhibited extreme physical activity or restricted weight gain as potential symptoms of an eating disorder. Although the physician was alerted several times to instances of weight loss, no participant was recommended to be withdrawn for this reason.

#### GWG Measurement Visits

The secondary outcomes included GWG from baseline to the final data collection point before delivery (32 weeks’ and 36 weeks’ gestation). At all measurement visits, participants’ weights were recorded in kilograms on a calibrated research-grade scale in duplicate, with the participants wearing light clothing and no shoes. In addition to the measured weight at baseline, participants also reported their preconception weight. Participants’ GWG goals were calculated based on self-reported preconception BMI. There is strong concordance between self-reported preconception weight and measured pregravid weight [46]. The weight at week 36 was the default outcome, except for participants who delivered earlier than 36 weeks, in which case their weight at week 32 was used. This approach is an attempt to have a final observation of GWG for all participants, regardless of whether they delivered earlier than 36 weeks.

### Power Estimates

This is a feasibility study, the primary purpose of which is to gather data that would evaluate the feasibility of recruitment, randomization, and retention, as well as treatment engagement and intervention satisfaction [47]. An additional aim of this study is to pilot the intervention components, consistent with the preparatory stage of the multiphase optimization strategy framework [48]. The trial was not powered to detect a significant difference among the conditions on treatment engagement or GWG.

### Statistical Analysis

We described characteristics of the sample using counts and percentages for categorical data and means and SDs for continuous data. We used generalized linear models with log link function and variance function proportional to the mean for analyzing the 2×2×2 factorial design for 2 outcomes: days of self-weighing and mean of each participant’s weekly physical activity minutes. The model included main effects for the 3 intervention components (self-weighing, weight goal, and physical activity goal) and all 2- and 3-factor interactions for constructing 8 conditions in the 2×2×2 factorial analysis. Because of the small sample size and our primary interest in the main effects, the analysis focused on reporting the main effects. The reference levels were loss incentive for self-weighing, overall GWG goal, and no physical activity goal. Participants’ GWG values according to the guidelines as an ordinal variable (below, within, and exceeded) were not included in this analysis because of the small sample size. We conducted 2-tailed *t* tests to test the null hypothesis of no difference in the incentive amounts received by component and overall. Minutes of physical activity were calculated based on days for which data were transmitted. All analyses were implemented using R (version 4.0.2; The R Foundation for Statistical Computing).

## Results

### Recruitment and Retention

A total of 41 participants indicated interest in the study over the 6-month recruitment period and completed the informed consent procedure, but 1 (2%) participant refused to participate after completing the informed consent process. In all, 40 participants were randomized; however, 1 (3%) participant was immediately withdrawn because of a staff member’s error in assessing this participant’s age eligibility (she was aged <18 years). This participant was not oriented to her condition and did not receive any intervention. Of the 39 participants who were oriented to their condition and received the intervention, 35 (90%) completed the follow-up data collection visit at 32 weeks’ and 36 weeks’ gestation; 1 (3%) participant delivered before the 36-week visit, and her 32-week weight was used. Among the 4 participants who did not complete the follow-up data collection visit, 1 (25%) relocated, 1 (25%) miscarried, 1 (25%) delivered her baby before the 32-week visit, and we were unable to contact 1 (25%) participant.

As can be seen in Table 4, the participants predominantly identified as Black or African American (24/39, 62%) and non-Hispanic (36/39, 92%). Among the 39 participants, for 12 (34%), this was their first pregnancy. Of the 39 participants, 30 (77%) were not married, 27 (70%) had at least some college education, 29 (74%) were employed, and 29 (74%) had an annual household income of less than US $50,000. With regard to managing GWG, of the 39 participants, 32 (82%) believed that the lottery-based daily self-weighing incentive would work better to help them and 31 (80%) believed that the monthly GWG goals would work better to help them. The participants were successfully recruited in the first trimester (mean gestational weeks at enrollment 9.6, SD 1.8), and they participated in the program for between 24 and 30 weeks.

**Table 4:** Sample characteristics (N=39).

				Values
Age at enrollment, mean (SD)				29.1 (12.5)
Gestational weeks at enrollment, mean (SD)				9.6 (1.8)
Number of children in household, mean (SD)				1.1 (0.9)
**Prepregnancy BMI categories, n (%)**				
	Normal weight (BMI 18.5-24.9 kg/m^2^)		17 (44)	
	Overweight (BMI 25-29.9 kg/m^2^)		13 (33)	
	Obese (BMI ≥30 kg/m^2^)		9 (23)	
Hispanic ethnicity, n (%)				3 (8)
**Race, n (%)**				
	White		10 (26)	
	Black or African American		24 (62)	
	Unknown or multiple races		5 (13)	
**Marital status, n (%)**				
	Married		9 (23)	
	Never married		18 (46)	
	A member of an unmarried couple		12 (31)	
**Education attainment, n (%)**				
	Some high school		2 (5)	
	High school graduate or GED^a^		10 (26)	
	Some college or technical school		20 (51)	
	College, 4 years or more (college graduate)		7 (18)	
**Employment status, n (%)**				
	Employed for wages		29 (74)	
	Self-employed		2 (5)	
	Out of work		2 (5)	
	Does not work outside the home		1 (3)	
	Student		4 (10)	
	Missing		1 (3)	
**Annual household income (US $) n (%)**				
	≤24,999		18 (46)	
	25,000-49,999		11 (28)	
	50,000-74,999		4 (10)	
	≥75,000		5 (13)	
	Missing		1 (3)	
**Which type of incentive do you think would work better for you to manage gestational weight gain?, n (%)**				
	**Daily self-weighing**			
		Lottery	32 (82)	
		Loss	7 (18)	
	**Weight gain goals**			
		Monthly weight gain goal	31 (80)	
		Overall weight gain goal	8 (20)	

^a^GED: General Educational Development.

### Intervention Satisfaction

Participants were generally quite positive about the intervention components (Table 5), with a mean score for each component of ≥3.8. Consistently, the mean score for the helpfulness and enjoyment aspect of the electronic scale was slightly higher than that for the other components; this positive view of self-weighing and weight tracking throughout pregnancy was also noted by 80% (28/35) of the participants in qualitative feedback regarding the aspects of the program that were most helpful to them. A participant stated, “Most helpful was having to weigh every day, it kept me in tune with where I was and what I needed to do differently.” In addition, in the qualitative responses, several participants indicated that it would have been helpful to have the option to receive SMS text messages rather than emails. A few participants also reported technical challenges, particularly with the e-scale. A participant noted, “I believe this is a great program for those who were already active prior to pregnancy,” perhaps reflecting the relatively low-intensity behavioral intervention for physical activity. Finally, participants indicated that additional features that might be helpful for this program would be dietary tracking and group meetings for peer support.

**Table 5:** Summary of intervention satisfaction (N=35).

			Values, mean (SD)
**Helpfulness for managing** **GWG^a^ (1=not at all helpful; 5=extremely helpful)**			
	Emails	4.4 (0.9)	
	Electronic scale	4.5 (0.9)	
	Fitbit	4.2 (1.0)	
**Usefulness for managing** **GWG** **(1=not at all useful; 5=extremely useful)**			
	Lottery-based self-weighing incentive	3.9 (1.1)	
	Loss-based self-weighing incentive	3.9 (1.0)	
	Monthly weight gain goal	4.1 (0.9)	
	Overall weight gain goal	4.0 (0.9)	
	Weekly physical activity incentive	3.8 (1.1)	
**How much did you enjoy the following? (1=not at all enjoyable; 5=extremely enjoyable) **			
	Using electronic scale	4.6 (0.7)	
	Using Fitbit	4.0 (1.2)	
	Frequency of receiving incentives	4.2 (0.8)	
	Frequency of receiving emails	4.1 (1.2)	
**How much did you enjoy receiving...incentives? (1=not at all enjoyable; 5=extremely enjoyable)**			
	Lottery-based self-weighing incentive	4.3 (1.1)	
	Loss-based self-weighing incentive	4.3 (1.1)	
	Monthly weight gain goal	4.3 (1.0)	
	Overall weight gain goal	4.2 (1.1)	
	Weekly physical activity incentive	4.1 (1.2)	
**Would you recommend...for managing** **GWG** **to a friend? (1=not at all likely; 5=extremely likely)**			
	Using an electronic scale	4.7 (0.7)	
	Using a fitness tracker	4.5 (0.9)	
	Setting a goal for weight gain	4.6 (0.6)	
	Setting a goal for physical activity	4.6 (0.7)	

^a^GWG: gestational weight gain.

### Intervention Adherence

The mean number of days that participants self-weighed on the study scale was 44.5 (SD 57.5) days.

Participants who were randomized to receive the loss incentive had a mean of 68.0 (SD 71.4) days of self-weighing compared with 19.8 (SD 18.8) days among those participants who were randomized to the lottery incentive. There was a significant main effect such that the mean difference in log means (or the log of the ratio of the means) was –1.05. The ratio of the means was 1/exp(1.05) = 1/2.86 = 0.35 (Table 6). In other words, participants who received the loss incentive, on average, had 2.86 times as many days of self-weighing as those who received the lottery incentive, or inversely the mean frequency of self-weighing with the lottery incentive was 0.35 times the loss incentive. Consistent with this greater adherence to self-weighing among those who received the loss incentive, participants in the loss incentive condition earned, on average, US $59.58, in self-weighing incentives in comparison with US $25.66 earned by those in the lottery incentive condition, although the amount that they could have earned in each condition was the same (US $112).

**Table 6:** Factorial main effects for all outcomes (N=39).

2×2×2 intervention components	Days of self-weighing			Mean of participants’ weekly PA^a^	
	Mean ratio (95% CI)	Estimate (SE)^b^	Mean ratio (95% CI)		Estimate (SE)
Self-weighing (reference=loss), lottery	0.35 (0.15-0.80)	–1.05^c^ (0.40)	5.38 (0.34-85.92)		1.68 (1.36)
Gestational weight gain goal (reference=overall), monthly	0.69 (0.30-1.58)	–0.36 (0.40)	0.24 (0.015-3.80)		–1.43 (1.36)
PA goal (reference=no incentives), incentives	1.26 (0.55-2.88)	0.23 (0.40)	0.79 (0.05-12.56)		–0.24 (1.36)

^a^PA: physical activity.

^b^Estimates and SEs are on the logarithmic scale; the mean ratio is obtained by exponentiating the main effect estimate.

^c^*P*<.01.

Participants, on average, had a relatively low level of activity, with a mean number of active minutes of 8.7 (SD 18.5) per week. Participants who were randomized to receive the physical activity incentive had a mean of 5.4 active minutes per week compared with 12.0 active minutes per week among those participants who were not randomized to receive physical activity incentives. However, there was no significant main effect. Of the 39 participants, 2 (5%) achieved the physical activity goal, and they received the US $3.50 incentive in just 1 week each.

Among the 35 participants who completed the follow-up data collection, 7 (20%) had weight gain below the GWG guidelines, 13 (37%) were within the guidelines, and 15 (43%) exceeded the guidelines. A similar proportion of individuals exceeded the guidelines, regardless of whether they were randomized to receive GWG incentives monthly (8/35, 44%) or overall (7/35, 41%). Of the 19 participants who were randomized to the monthly GWG incentive component, 8 (42%) received at least one monthly US $14 incentive (with a range of 1-5 monthly incentives received).

Participants earned, on average, US $43.05 (range US $0 to US $211) in total. Not surprisingly, participants who were within the guidelines earned more on average (US $91.27) compared with those who were below the guidelines (US $16.50) and those who exceeded the guidelines (US $19.87).

## Discussion

### Principal Findings

We demonstrated the feasibility of conducting a randomized controlled trial with a 2×2×2 factorial design at an obstetric clinic that provided financial incentives for self-weighing, physical activity, and GWG within the IOM guidelines. We successfully recruited participants early in their first trimester of pregnancy and retained 90% (35/39) of these participants for follow-up data collection in the third trimester. We were also successful in randomizing participants to this complex randomization scheme and providing the assigned intervention components. In addition, the intervention was well-received, with high satisfaction ratings from the participants for all the components and a particular focus on the helpfulness of self-weighing and weight tracking. The participants engaged in self-weighing approximately 7 days per month, in contrast to previous research indicating that regular self-weighing is uncommon among pregnant women without intervention [49]. However, there was low engagement in physical activity among these participants, regardless of whether they received an incentive for reaching the 150-minute physical activity goal.

On the basis of the projected clinic flow of 16 pregnancy confirmation appointments per month, we were successful in recruiting more than one-third of the patients per month, with more than 6 participants recruited to the study per month. Notably, all but 2 participants who indicated initial interest were eligible and interested in being randomized. In addition, retention was very good, with only 10% (4/39) of the participants not participating in follow-up data collection (including a participant who had a miscarriage and a participant who gave birth before the 32-week visit).

Contrary to previous literature that has reported that participants prefer incentive schemes *other* than lotteries [50-52], the participants in this study indicated that they believed that the lottery-based incentive for self-weighing would be more helpful for them in managing GWG than the loss-based incentive. Surprisingly, contrary to their stated preference, participants who were randomized to receive the loss-based incentive had significantly greater self-weighing engagement compared with those randomized to the lottery-based incentive. This finding is consistent with a recent study, which found that loss-based incentives were more effective in increasing step goal attainment than a lottery-based incentive [37].

Although regular physical activity is recommended for a healthy pregnancy [44], most women in general are physically inactive, particularly during pregnancy [53]. Consistent with these previous findings, we found that women in our sample had a very low level of physical activity overall. The physical activity incentives seemed to do little to increase physical activity. On the basis of a recent meta-analysis [54], more intensive intervention such as supervised exercise may be necessary for increasing physical activity during pregnancy.

This study is the first to offer both *process* incentives (for behaviors) and *outcome* incentives (for achieving monthly or overall GWG), within the context of gestational weight management. However, there were no clear trends in this study as to whether an incentive for achieving a monthly or an overall GWG goal was associated with gaining weight within the IOM guidelines. A similar proportion of participants in this sample (13/36, 37%) gained weight within the guidelines compared with the national prevalence (32.1%) [10]. Although this study was not powered to detect differences in GWG recommendation adherence, this finding is consistent with previous research indicating that incentives did not increase adherence to GWG recommendations [22]. Should the lack of impact of financial incentives on GWG outcomes be confirmed in future fully powered research, it may be that financial incentives are more powerful at encouraging behaviors associated with GWG than GWG outcomes themselves. In that case, it would then be essential to determine whether the costs to achieve certain behaviors in pregnancy (such as self-weighing) are beneficial independently. For example, it is possible that incentives for self-weighing may not lead to optimal GWG (as a categorical variable), but incentives for self-weighing may reduce the mean amount of GWG [55] and thus reduce the likelihood of negative maternal health outcomes.

This study includes several notable strengths and weaknesses. First, the recruited sample was quite diverse in race, income level, and BMI category and, thus, included many individuals who often are not included in research [56,57]. However, this study had a small sample to determine feasibility and recruited from only 1 obstetric clinic; thus, future fully powered research is necessary. In addition, the incentive strategies and amounts used in this study were only a few of the many possible approaches, and it is possible that there are more effective alternatives that should be tested. A further limitation is that the data reported here and the incentives provided are based on the data received by the research team and not necessarily all of the data sent by the participants. This is an important distinction because 5% (2/39) of the participants reported having technical problems in the program evaluation. It is also possible that the low levels of physical activity were due to undetected difficulties in transmitting physical activity data. In addition, although we did not report all the costs of the intervention, the unreported costs (including the costs of the scale and the Fitbit activity tracker) were the same for all participants.

This feasibility study provides valuable information for future research, including the expected rate of clinic-based recruitment per physician for a low-intensity intervention, the high rate of retention with convenient clinic-based data collection, and expected adherence and costs for a financial incentive–based intervention. In future research, given our few unresolved technical difficulties, it may be important to set up a telephone visit after a few days to ensure that participants are able to use the technology in their home environment and test using multiple modalities (SMS text messages, telephone, and email) for investigating potential technical problems. The findings from this study also indicate enthusiasm for self-weighing among pregnant women as a strategy for gestational weight management, which may be important in designing future studies. These results also may indicate that preferences for an intervention strategy may not translate into the more effective strategy, which has also been demonstrated in other research [58]. Our findings indicate that although the loss-based incentives may not be perceived as more effective at baseline, they are a promising strategy for increasing self-weighing behaviors. Finally, this feasibility study suggests that more intense intervention strategies (such as meal replacements, motivational interviewing or problem-solving sessions, calorie goals, and dietary self-monitoring with feedback from an interventionist) may be necessary for increasing moderate physical activity among pregnant women and adherence to the GWG guidelines [59,60]. For example, future research in this area could be a factorial experiment that examines the combination of outcome and behavioral incentives with more intense intervention strategies.

### Conclusions

This pilot randomized controlled trial indicates that a financial incentive–based pragmatic intervention is feasible and acceptable for pregnant women for promoting self-weighing, physical activity, and healthy GWG. Participants were successfully recruited early in their first trimester of pregnancy and retained for follow-up data collection in the third trimester. Participants demonstrated promising engagement in self-weighing, particularly with loss-based incentives, and reported finding the self-weighing especially helpful. This study supports further investigation of pragmatic, clinic-based interventions for healthy GWG.

## Appendix

Multimedia Appendix 1 Recruitment brochure.

Multimedia Appendix 2 Study handout: strategies for achieving healthy gestational weight gain.

Multimedia Appendix 3 Example email messages for each intervention condition.

Multimedia Appendix 4 CONSORT-eHEALTH checklist (V 1.6.1).

## Abbreviations

GWG: gestational weight gain
IOM: Institute of Medicine
