# Supplementary material for: A Pragmatic Intervention Using Financial Incentives for Pregnancy Weight Management: Feasibility Randomized Controlled Trial
Source: JMIR Form Res. 2021 Dec 24;5(12):e30578. doi: 10.2196/30578 (PMC8742213; doi:10.2196/30578)
Supplement: Multimedia Appendix 1 [file formative_v5i12e30578_app1.pdf]

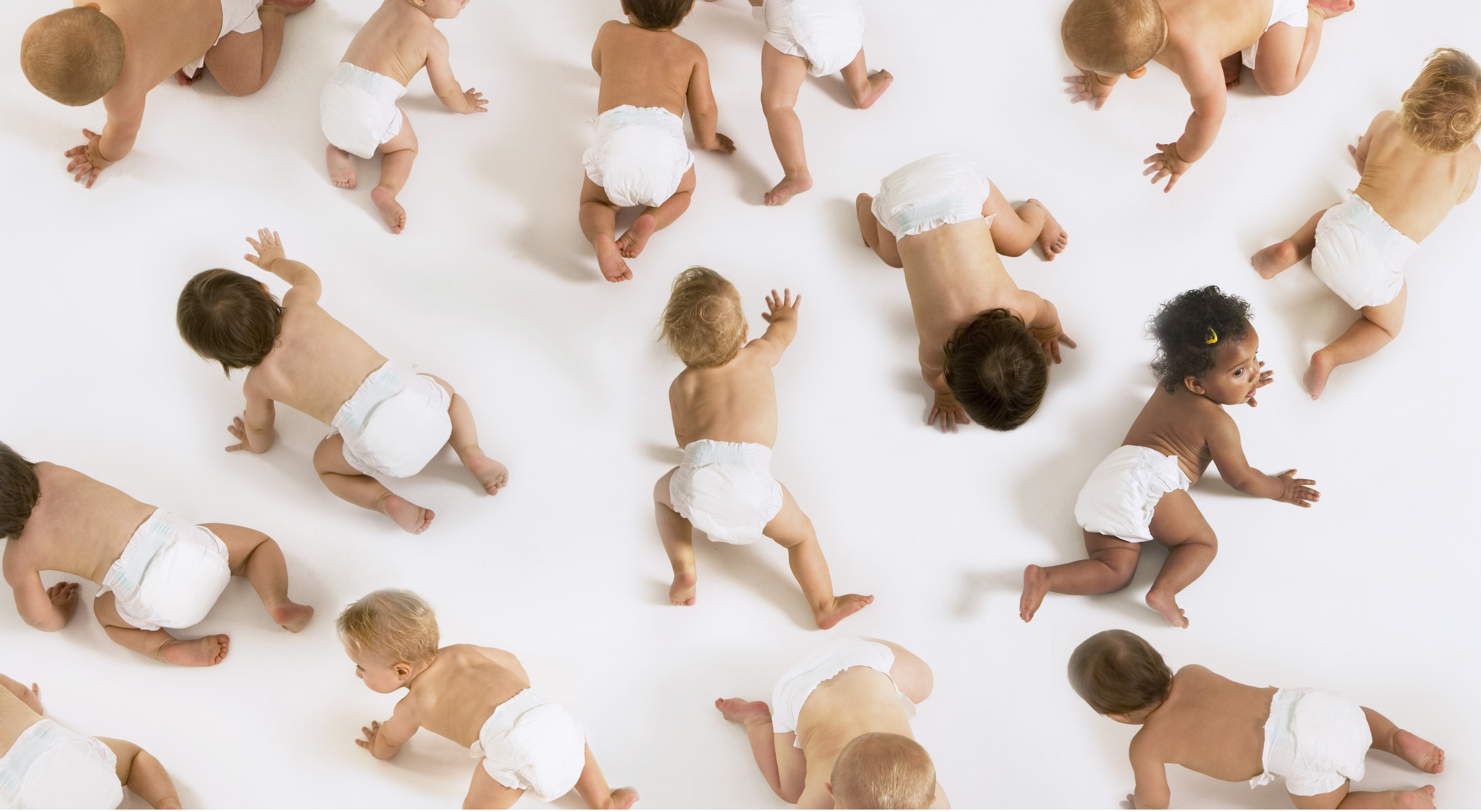

Bloom

RESEARCH STUDY

Want to make a healthy pregnancy a bit more fun?!

INTERESTED? PLEASE CONTACT US AT:

DR. REBECCA KRUKOWSKI
66 N PAULINE ST
MEMPHIS TN, 38163

OFFICE: 901-448-2426
STUDY PHONE: 901-448-2716
BLOOMUT@UTHSC.EDU

THE UNIVERSITY OF TENNESSEE HEALTH SCIENCE CENTER.

ABOUT THIS STUDY

Gaining more weight than the Institute of Medicine's recommendations during pregnancy increases risk for negative health outcomes for both you and your baby.

But there are so many do's and don'ts during pregnancy-- can financial incentives make having a healthier pregnancy fun?!

Bloom can help you monitor your pregnancy weight gain and exercise for up to 9 months.

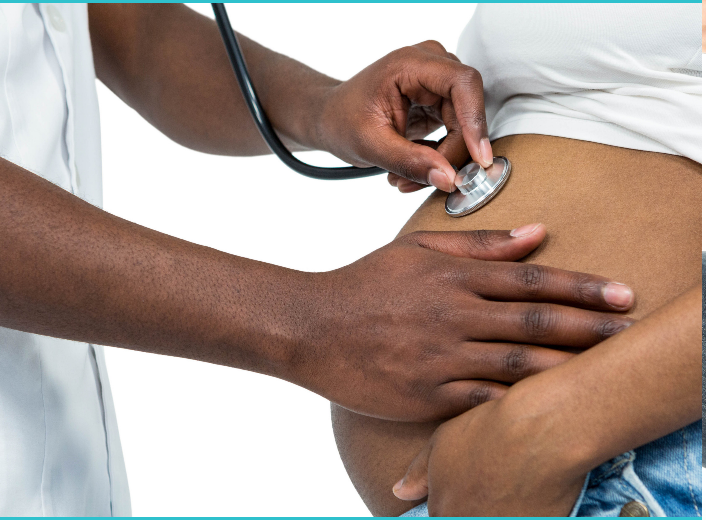

You are eligible if you:

- ☑ Are less than 12 weeks pregnant
- ☑ Are 18 years of age or older
- ☑ Have regular access to internet

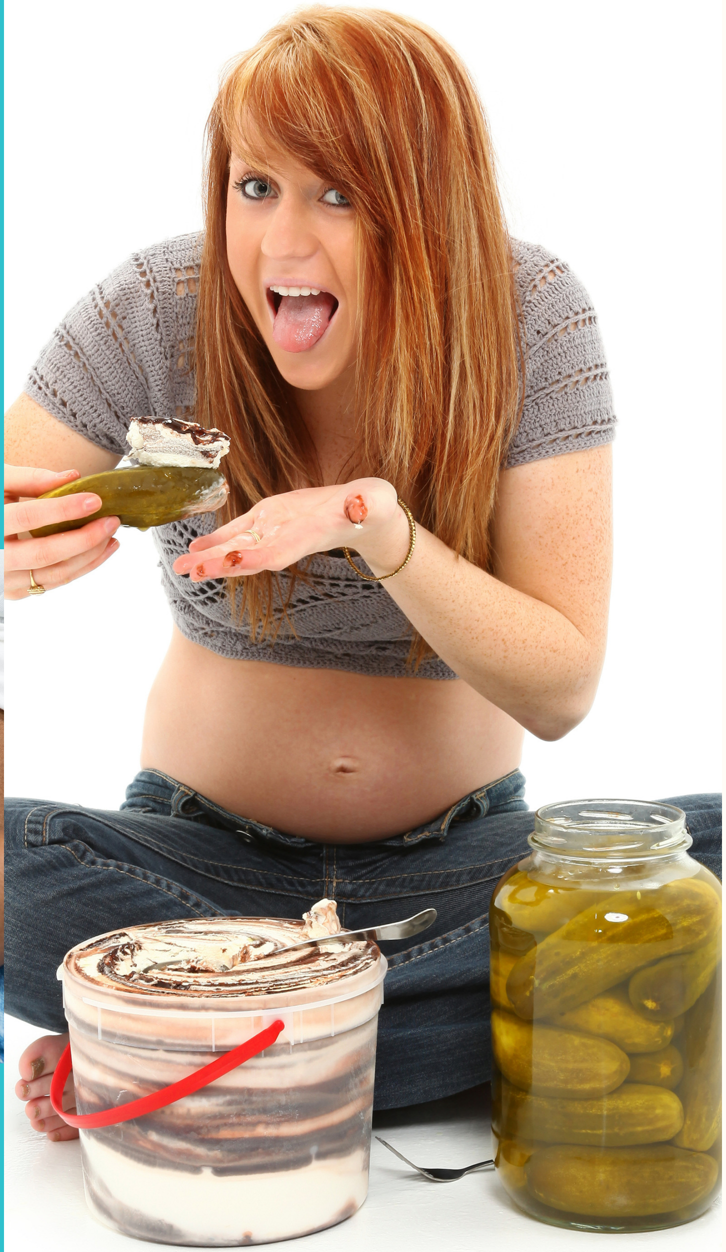

STUDY COMPONENTS:

Participants are randomized into 1 of 8 conditions with the possibility of incentives for:

- WEIGHING YOURSELF DAILY
- ACHIEVING THE INSTITUTE OF MEDICINE'S MONTHLY OR OVERALL WEIGHT GOAL
- ACHIEVING 150 MINUTES OF PHYSICAL ACTIVITY EACH WEEK AS RECOMMENDED

FOLLOW-UP DATA COLLECTION VISITS:

- AT 32 AND 36 WEEKS GESTATION
- LESS THAN 10 MINUTES
- COMPENSATION FOR YOUR TIME
