# Supplementary material for: A Pragmatic Intervention Using Financial Incentives for Pregnancy Weight Management: Feasibility Randomized Controlled Trial
Source: JMIR Form Res. 2021 Dec 24;5(12):e30578. doi: 10.2196/30578 (PMC8742213; doi:10.2196/30578)
Supplement: Multimedia Appendix 2 [file formative_v5i12e30578_app2.pdf]

Strategies to Achieve Your Healthy Pregnancy Goals and Earn Bloom Incentives!

Track

Track your weight and physical activity with the scale and Fitbit. You might find tracking your diet helpful with LoseIt! or MyFitnessPal.

Measure

You can't know how much you are eating unless you measure it-- try measuring spoons and cups or a food scale!

Set Goals

Make your goals REALLY specific, measureable, broken down into steps, and determine how long you will try it before you re-evaluate.

Manage Hunger

Aim for foods that have lots of water, fiber, and/or air (e.g., fruits, vegetables, broth-based soup, oatmeal, popcorn) which help fill you up for not very many calories.

Choose Alternatives

Swap for healthier options. For example, choose baked chicken instead of fried chicken, or order fruit or a salad instead of fries.

Find Solutions

Challenges always come up, and when they do:

1. Describe the problem
2. Brainstorm options
3. Pick a solution
4. Make a plan
4. Try it!

Liquid Calories

It can be easy to forget about calories in juice, coffee drinks, sweet tea, or soda. Be aware!

Support

Identify family members or friends who can help you achieve your goals. For example, they may cheer you on, provide accountability or exercise with you.

Bloom

RESEARCH STUDY
