# Supplementary material for: A Pragmatic Intervention Using Financial Incentives for Pregnancy Weight Management: Feasibility Randomized Controlled Trial
Source: JMIR Form Res. 2021 Dec 24;5(12):e30578. doi: 10.2196/30578 (PMC8742213; doi:10.2196/30578)
Supplement: Multimedia Appendix 3 [file formative_v5i12e30578_app3.docx]

Supplemental Materials 3: Example messages for each intervention condition

Self-weighing lottery-based incentive example messages

*Weighed and matched*: “I knew you’d be up for the challenge-- you weighed yourself yesterday! We picked your lucky number, so you won [AMOUNT] from the lottery!”

*Weighed and not matched*: “Great job stepping on the scale yesterday! Alas, the number drawn was [WINNING_NUMBER] and your number is [YOUR_NUMBER]. So, you did not win yesterday. Have you already stepped on the scale today for another chance to win?”

*Did not weigh and matched*: “Knowing your weight is helpful to maintain a healthy weight gain throughout your pregnancy. Let your weight be your guide. You would have won [AMOUNT] had you weighed yourself yesterday.”

*Did not weight and not matched*: “We didn't see a weight on the Withings scale from you yesterday. (Check in with us to problem solve regarding the technology if you did weigh yourself on the scale we gave you.) The number drawn was [WINNING_NUMBER] and your number is [YOUR_NUMBER].”

Self-weighing loss-based incentive example messages

*Weighed*: “Hotdiggity! You get to keep $0.50 for the day because you weighed yourself on the Withings scale! Monitoring your weight closely can help you achieve a healthy weight gain during pregnancy!”

*Did not weigh*: “Because you did not weigh yourself on the Withings scale yesterday, you lost $0.50. What were the barriers to weighing yourself yesterday? How might you overcome those barriers today?”

Monthly GWG goal incentive example messages

*Within the recommended range*: “Wonderful job working toward a healthy pregnancy for both you and your baby! You earned $14 for staying within the weight gain goal this month.”

*Not within the recommended range*: “Your weight gain was not within the recommended range last month, so you did not earn $14 this month. A slow and steady weight gain will help you have a healthy pregnancy and baby. This month you have another chance!”

Weekly physical activity goal incentive example messages

*Met the exercise goal*: “You earned $3.50 last week because you reached the 150-minute exercise goal last week that is recommended by the American College of Obstetrics and Gynecology! Exercise is great for both you and your baby!”

*Did not meet the exercise goal*: “Exercise helps you sleep better—what pregnant woman does not want more or better sleep?! Although you did not earn $3.50 for reaching the exercise goal last week, this week is a new week. How might you fit in exercise this week AND maybe sleep better?”
